# S29. CONCORDANCE BETWEEN SELF-REPORT AT INTERVIEW-BASED RATINGS OF PSYCHOTIC EXPERIENCES IN PRE-ADOLESCENCE AND ASSOCIATED PSYCHOPATHOLOGY

**DOI:** 10.1093/schbul/sby018.816

**Published:** 2018-04-01

**Authors:** Martin K Rimvall, Steffie Gundersen, Lars Clemmensen, Charlotte Ulrikka Rask, Anne Mette Skovgaard, Frank Verhulst, Jim van Os, Pia Jeppesen

**Affiliations:** 1 Child and Adolescent Mental Health Center, Mental Health Services, The Capital Region of Denmark; 2 Aarhus University Hospital; 3 University of Copenhagen; 4 Maastricht University Medical Centre

## Abstract

**Background:**

Self-report measures are often used to assess psychotic experiences (PE) in large-scale epidemiological samples, and have contributed substantially to our knowledge on PE. However, different self-report PE (PE-S) measures have yielded particularly wide-spread prevalence-estimates of PE ranging from 21–66 % in 7–13 year old children, whereas interview based measures of PE (PE-I) vary less (10 -23 %). Especially PE-S have been criticized for being over-inclusive and not capturing the essence of low-grade psychosis. The current study is the first large-scale study to examine the psychometric properties of a PE-S measure in children, and the first to compare the clinical correlates of PE-S and PE-I in the same sample.

**Methods:**

As part of the general population Copenhagen Child Cohort 2000 studies, 1571 children aged 11–12 years were independently assessed for both PE-I and PE-S. PE-I were assessed by trained professionals with 22 items on hallucinations and delusions from the Kiddie Schedule for Affective Disorders and Schizophrenia present and life-time version (Kiddie-SADS-PL). PE-S were assessed by 10 questions covering hallucinations, delusions and subjective thought disturbances ever in life, forming a new section of the diagnostic interview, the Development and Well Being Questionnaire (DAWBA).

We assessed the psychometric properties of PE-S, using PE-I as the “gold-standard”. We analyzed the association between PE-S and emotional and neurodevelopmental DSM-IV disorders of the child as well as a history of psychotic disorders in 1st degree family members. Both have previously been examined in previous studies of the current cohort, and were significantly associated with PE-I.

**Results:**

The prevalence of PE-S was higher compared to PE-I, 28.1% and 10.2% respectively. The predictive values of any type of PE-S for any PE-I were: sensitivity = 73.8%, specificity = 77.1%, positive predictive value = 26.8% and negative predictive value = 96.3%.

The association between PE and mental health disorders and a family history of psychotic disorders yielded slightly lower odds ratios (OR) for PE-S compared to PE-I. However, the associations remained statistically significant and had overlapping confidence intervals: For any emotional or neurodevelopmental DSM-IV disorder: PE-I OR 3.3 (CI95% 2.3–4.8) and PE-S OR 2.7 (CI95% 2.1–3.6), for a 1st degree family history of psychotic disorder; PE-I OR 4.9 (CI95% 1.9–12.5) and PE-S OR 2.6 (CI95% 1.1–6.3).

**Discussion:**

PE-S were almost 3 times more likely to be reported, compared to observer-rated PE-I. However, the associations with unfavorable clinical correlates were only slightly attenuated for PE-S when compared to PE-I. The study confirmed that PE-S are clinically relevant, and the DAWBA-section proved valuable as a screening tool for PE in the pre-adolescent general population.

